# A systematic review and network meta-analysis of pharmaceutical interventions used to manage chronic pain

**DOI:** 10.1038/s41598-023-49761-3

**Published:** 2024-01-18

**Authors:** Ashish Shetty, Gayathri Delanerolle, Heitor Cavalini, Chunli Deng, Xiaojie Yang, Amy Boyd, Tacson Fernandez, Peter Phiri, Arun Bhaskar, Jian Qing Shi

**Affiliations:** 1 University College London Hospitals NHS Foundation Trust, London, UK; 2 University College London, 235, Euston Road, London, NW1 2BU UK; 3 Pain Medicine, Cleveland Clinic London, London, United Kingdom; 4 Nuffield Department of Primary Care Health Sciences, University of Oxford, Oxford, OX3 7JX UK; 5 Southern Health NHS Foundation Trust, Southampton, SO40 2RZ UK; 6 Southern University of Science and Technology, Shenzhen, 518055 China; 7 School of Statistics and Mathematics, Yunnan University of Finance and Economics, Kunming, China; 8 National Centre for Applied Mathematics Shenzhen, Shenzhen, China; 9 University of Oxford, Oxford, UK; 10 Psychology Department, Faculty of Environmental and Life Sciences, University of Southampton, Southampton, SO17 1BJ UK; 11 Imperial College Healthcare NHS Trust, London, UK

**Keywords:** Neuropathic pain, Drug development

## Abstract

It is estimated 1.5 billion of the global population suffer from chronic pain with prevalence increasing with demographics including age. It is suggested long-term exposure to chronic could cause further health challenges reducing people’s quality of life. Therefore, it is imperative to use effective treatment options. We explored the current pharmaceutical treatments available for chronic pain management to better understand drug efficacy and pain reduction. A systematic methodology was developed and published in PROSPERO (CRD42021235384). Keywords of opioids, *acute pain*, *pain management*, *chronic pain*, *opiods*, *NSAIDs*, and* analgesics* were used across PubMed, Science direct, ProQuest, Web of science, Ovid Psych INFO, PROSPERO, EBSCOhost, MEDLINE, ClinicalTrials.gov and EMBASE. All randomised controlled clinical trials (RCTs), epidemiology and mixed-methods studies published in English between the 1st of January 1990 and 30th of April 2022 were included. A total of 119 studies were included. The data was synthesised using a tri-partied statistical methodology of a meta-analysis (24), pairwise meta-analysis (24) and network meta-analysis (34). Mean, median, standard deviation and confidence intervals for various pain assessments were used as the main outcomes for pre-treatment pain scores at baseline, post-treatment pain scores and pain score changes of each group. Our meta-analysis revealed the significant reduction in chronic pain scores of patients taking NSAID versus non-steroidal opioid drugs was comparative to patients given placebo under a random effects model. Pooled evidence also indicated significant drug efficiency with Botulinum Toxin Type-A (BTX-A) and Ketamine. Chronic pain is a public health problem that requires far more effective pharmaceutical interventions with minimal better side-effect profiles which will aid to develop better clinical guidelines. The importance of understanding ubiquity of pain by clinicians, policy makers, researchers and academic scholars is vital to prevent social determinant which aggravates issue.

## Introduction

Chronic non-cancer pain conditions are prevalent, highly debilitating and have high cost implications to health and social care. These conditions affect patients, their families and society at large, impacting 20% of the global population^1^. The prevalence of pain conditions among females of all ages appears to be increasing^2^. Complexities around diagnosis and treatment of chronic pain conditions have meant that there is a paucity of standardised clinical guidelines that could potentially improve the clinical practice landscape, globally.

Convalescent periods for many chronically ill patients can be protracted and daunting. This may be especially true where pain medication has been used in the long term^3^. Long-term exposures to chronic pain coincide with mental health and wellbeing, exacerbating patient-reported outcomes such as sleep disturbances, depression, dependence and morbidities such as myalgia and fatigue^4^. Better understanding of long-term implications requires consideration of “life-course approaches” and at present, this could evolve further within pain medicine epidemiology^5^.

Increases in chronic pain conditions contributes to higher healthcare costs towards clinical management of patients and also reduced levels of productivity for employers^6^. This may be partly due to increases in opioid use within this population of patients, often reducing their capacity to conduct normal working hours. Current clinical guidelines recommend non-invasive pain management options as a first-line treatment among non-cancer patients in particular, although overdose, dependency and mortality due to opioid use has consistently increased over time^7,8^. It was reported that global opioid use has doubled between 2001 and 2003 to 2011 and 2013 to 7.35 billion daily doses per year^9,10^.

It is particularly important to develop evidence-based guidelines specific to each condition, with flexible pain medication use as a single regimen or a combination of treatments that could improve the overall quality of life of these patients^11,12^. The premise to increase the strength and frequency of pain medications is in general based on disease burden i.e., progression of symptoms and patients reported symptoms^4^.

We have designed the POP project as the initial step to conduct exploratory work on pharmaceutical management of chronic pain. With the rising need for comparative effectiveness research, increasingly more systematic reviews focus on evaluating the relative efficacy and acceptability of drugs and therapeutic interventions^3,13^. However, some of the interventions for long-term conditions are not available for clinical practice and there are several options with varying efficacy even within a specific class of interventions^14^.

## Methods

We developed a wide systematic methodology and published this as a protocol with multiple research questions in the first instance in PROSPERO (CRD42021235384). Data from studies meeting the inclusion criteria were extracted and Pairwise Meta-Analysis with random and fixed effects models was carried out. Pooled mean difference (MD) together with 95% confidence intervals (CIs) are reported overall and for sub-groups. By combining the direct and indirect comparisons between different interventions, Network Meta-Analysis was conducted to explore the relative treatment effects among all the drugs included in our analysis.

### Aims

The aims of the study was to explore the prevalence of treatments of effects in chronic pain based on pharmaceutical treatments.

### Search strategy

The search strategy used key words of *chronic pain*, *opioids*, *acute pain*, *pain management*, *opiods*, *NSAIDs, analgesics* across multiple databases (PubMed, Science direct, ProQuest, Web of science, Ovid Psych INFO, PROSPERO, EBSCOhost, MEDLINE, ClinicalTrials.gov and EMBASE).

### Eligibility criteria

**Figure 1:**
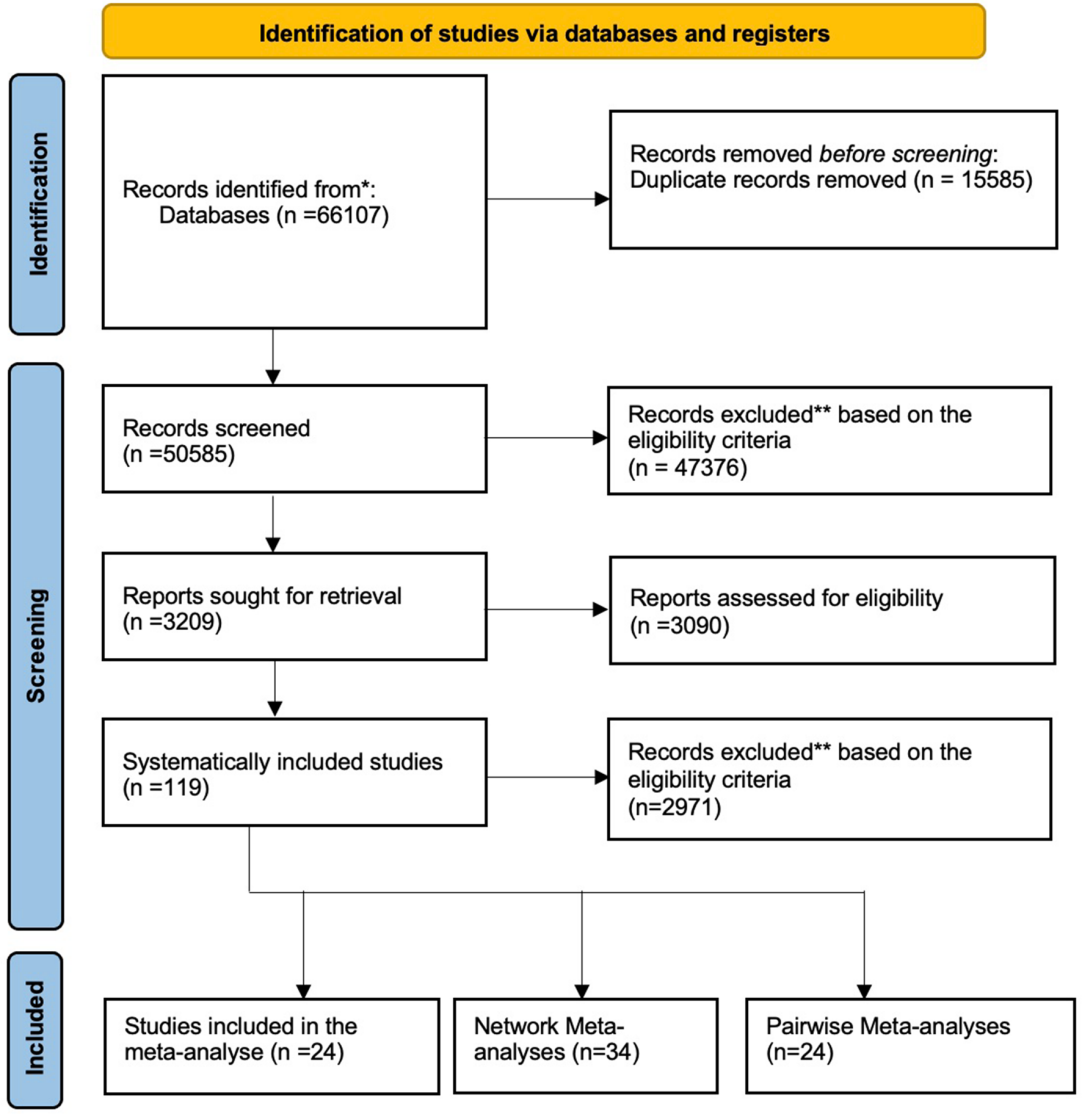
PRISMA 2020 flow diagram for new systematic reviews which included searches of databases and registers only^15^.

All randomised controlled clinical trials (RCTs), epidemiology and mixed-methods studies reporting the use of pain medication for non-cancer chronic pain conditions published in English between the 1st January 1990 and 30st April 2022 were included. Opinions, commentaries and editorials were excluded (Fig. 1).

### Data extraction

Participants included in the study populations had chronic non-cancer pain conditions. All studies reporting drug efficacy were extracted by way of the interventions, measures of tool and numeric results. An extraction template specific to the objectives of the study was developed. Sub-studies were extracted from the same clinical trials with different duration periods.

Data was extracted by two investigators and any disputes for eligibility was discussed and agreed with the Chief Investigator of the study. All studies included within the analyses were independently reviewed.

### Outcome measures

Outcomes were reported as mean, median, standard deviation and confidence intervals. Mean and Standard deviation (SD) were extracted as the main outcomes including pre-treatment pain scores at baseline, post-treatment pain scores and pain score changes of each group.

Multiple pain assessments for confirming a clinical diagnosis, severity and progression of chronic pain were identified. These include VAS (visual analogue scale, 0–10 or 0–100), NRS (11-point numeric rating scale, 0–10), BPI (Brief Pain Inventory interference scale, 0–10), MPQS (McGill Pain Questionnaire-Short Form (Sensory and Affective subscales, VAS intensity measure, 0–10), VRS (verbal rating scale, 0–10), NIH-CPSI (National Institutes of Health Chronic Prostatitis Symptom Index, pain scores, 0–21), PI (pain intensity on a 20-point scale, 0–20).

As most widely used tools for assessing pain such as VAS, NRS, VRS, use a 11-point numeric rating scale from 0 to 10, the following standardisation formula was used to unify all pain scores into the same scale:

$$\mathrm{Scaled \, Pain \, Score }=\mathrm{ Original \, Pain \, Score }* \frac{10}{\mathrm{Scale \, Range}}$$

As all outcomes of interest were continuous, the calculation based on pain scores was performed by using mean differences (MD) with a 95% confidence interval (CI) to report the effects between the group comparisons.

### Exposures

The exposures of interest were selected based on the key features of pharmacological management used to treat non-cancer chronic pain, including and not limited to a pain condition being the primary or the secondary condition. Neurological and psychological symptoms leading up to the use of pharmaceutical use within the included population were also considered.

### Statistical analysis plan

A meta-analysis, pairwise meta-analysis (PMA) and Network meta-analysis (NMA) were used to compare all treatments used in managing non-cancer chronic pain. The fundamental difference between them is that PMA produced only one estimate of pooling effects from the selected pair of interventions, while the NMA produced multiple comparative estimates of pooling effects by connecting all alternative interventions^16^.

We incorporated direct and indirect treatment comparisons within the NMA providing greater statistical precision compared to a PMA. Rankings of a set of drugs or combined interventions for assessing chronic pain with respect to their efficacy was calculated based on the network models. Homogeneity and Consistency were tested to see if the assumptions in NMA were violated. The overall pharmaceutical efficacy of extracted studies was produced by pooling all treatment effects. PMA was also used on studies with the same drug as the treatment group to see the specific drug efficacy.

${{\text{I}}}^{2}$ and p-value were commonly used to detect statistical heterogeneity. A value of ${{\text{I}}}^{2}$ larger than 50% with a much smaller p-value indicates strong heterogeneity. Correspondingly, ${{\text{I}}}^{2}$ less than 50% with a large p-value indicates fairly weak heterogeneity^17^. A random effects model was chosen when there was high heterogeneity, whereas a fixed effects model was used if weak or no heterogeneity was detected^18^. Due to the presence of high heterogeneity, subgroup analyses were carried out to identify the sources. To assess the robustness of the pooled results within the PMA, a sensitivity analysis was completed. Publication bias was evaluated with funnel plots and Egger tests. The statistical analyses were produced by R and packages were used to provide outputs in compliance with best practice and reporting guidelines^19^.

## Results

Of the 119 systematically included studies (Table 1) with 17,708 participants, 24 studies were used in the meta-analysis and 34 within the NMA to build a connected network.

**Table 1:** Characteristics of the studies included in systematic review.

Study ID	Authors	Publication year	Study type	Pain type	Intervention	Sample size	Mean age	Country	Included for MA	Included for NMA
1	Weizman et al.	2018	P–C, RCT	Chronic-pain	THC	17	33.3	Israel	No	No
2	Krebs et al.	2018	RCT	Back, Arthritis, Chronic-pain	Opioid	240	56.8	USA	No	No
3	AbdelHafeez et al.	2019	Double-blind, P–C, RCT	Chronic-pain	Gabapentin	60	32.7	UK	Yes	Yes
4	Bushey et al.	2021	RCT	Chronic-pain	Opioid	241	37	USA	No	No
5	Bruehl et al.	2021	Double-blind, P–C, RCT, Crossover	Low-back, Chronic-pain	Morphine + Naloxone	191	36.5	USA	No	No
6	Worley et al.	2015	RCT	Chronic-pain	Buprenorphine/Naloxone	149		USA	No	No
7	Dindo et al.	2018	Single-blinded, RCT	Postsurgical, Chronic-pain	ACT	76	62.2	USA	No	No
8	Hruschak et al.	2019	Single-blinded, RCT	Chronic-pain	IPGT	30	53.9	USA	No	No
9	Azevedo et al.	2013		Chronic-pain	Opioid	2213	45	Portugal	No	No
10	Gudin et al.	2020	Open-label, P–C, Uncontrolled	Low-back, Noncancer, Chronic-pain	NKTR-181	402	52	USA	No	No
11	Stahl et al.	2019	RCT	Low-back, Chronic-pain	Venlafaxine	209	69.6	USA	No	No
12	Schliessbach et al.	2018	Double-blind, P–C, RCT	Low-back, Chronic-pain	Imipramine	50	54.4	Switzerland	No	No
13	Mohamed et al.	2016	Double-blind, RCT	PostsurgicalNeuropathic, Cancer, Chronic-pain	Morphine	90	50.43	Egypt	No	No
14	Schliessbach et al.	2018	P–C, RCT	Low-back, Chronic-pain	Oxycodone + Imipramine + Clobazam	98	55	Switzerland	No	Yes
15	Hermans et al.		Double-blind, P–C, RCT, Crossover	Arthritis, Chronic-pain	Naloxone	31	39.8	Belgium	No	No
16	Todorov et al.	2005		Chronic-pain	Gabapentin + Tiagabine	91	42	USA	No	Yes
17	Sadatsune et al.		Double-blind, P–C, RCT	Chronic-pain	Gabapentin	40	51.5	Brazil	No	No
18	Edwards et al.	2016	RCT	Back, Chronic-pain	Opioid	31	49	USA	No	No
19	Katz et al.	2011	Double-blind, P–C, RCT	Low-back, Chronic-pain	Naproxen + Tanezumab	129	52.1	USA	No	No
20	Hayek et al.	2021	Double-blind, RCT, Crossover	Chronic-pain	Opioid + Bupivacaine	16	63.1	USA	No	No
21	Schliessbach et al.	2017	Double-blind, P–C, Crossover	Back, Chronic-pain	Clobazam	49	54.3	Switzerland	No	Yes
22	Bruehl et al.	2004	Double-blind, P–C, RCT, Crossover	Low-back, Noncancer, Chronic-pain	Opioid	28	37.3	USA	No	No
23	Kim et al.	2018	Double-blind, RCT	Postsurgical, Chronic-pain	Nefopam	58	40	South korea	No	No
24	Eisenach et al.	2010	Double-blind, P–C, RCT, Crossover	Chronic-pain	Ketorolac	15	44		No	No
25	Rauck et al.	2014	Single-blinded, RCT, Crossover	Chronic-pain	Adenosine/Clonidine	22	44	USA	No	No
26	Buchheit et al.	2019	Double-blind, P–C, RCT	Postsurgical, Chronic-pain	Valproate	128	57	USA	No	No
27	Papadokostakis et al.	2005		Back, Chronic-pain	Calcitonin	110	65	Greece	No	No
28	Gould et al.	2020	Double-blind, 4-arm, RCT	Back, Chronic-pain	Desipramine	141	51.5	USA	No	Yes
29	Schnitzer et al.	2016	Double-blind, P–C, RCT	Back, Chronic-pain	D-cycloserine	41	53.2	USA	No	No
30	Nenke et al.	2015	Double-blind, P–C, RCT, Crossover	Low-back, Noncancer, Chronic-pain	Hydrocortisone	26	71	Australia	Yes	Yes
31	Sopata et al.	2015	Double-blind, P–C, RCT	Chronic-pain	Opioid	100	62.1	Poland	No	No
32	Kendall et al.		Double-blind, P–C, RCT	Postsurgical, Chronic-pain	Lidocaine	148	48	usa	No	No
33	Hongo et al.	2015	RCT	Back, Chronic-pain	Risedronate + Elcatonin	45	70.9	Japan	No	No
34	Amr and Yousef	2010	Double-blind, RCT	Postsurgical, Chronic-pain	Venlafaxine + Gabapentin	150	45	Egypt	No	No
35	Pedersen et al.	2014	Double-blind, RCT	Chronic-pain	Codeine + Paracetamol	58	49	Norway	No	No
36	Choi et al.	2016	Double-blind, RCT	Postsurgical, Chronic-pain	Lidocaine	90	34	Korea	No	No
37	Bruehl et al.	2014	P–C, RCT	Back, Chronic-pain	Morphine + Naloxone	50	36.9	USA	Yes	Yes
38	Chrubasik et al.	2010	Double-blind, P–C, RCT	Chronic-pain	Capsicum	130	48.9	Germany	No	No
39	Schliessbach et al.	2017	Double-blind, P–C, RCT, Crossover	Back, Chronic-pain	Oxycodone	50	55	Switzerland	No	Yes
40	Bruehl and Chung	2006	Double-blind, P–C, RCT, Crossover	Low-back, Chronic-pain	Naloxone	119	35.1	USA	No	No
41	Bruehl et al.	2013	Double-blind, P–C, RCT, Crossover	Low-back, Chronic-pain	Naloxone + Morphine	76	37.9	USA	No	No
42	Burns et al.	2017	Double-blind, P–C, RCT	Low-back, Chronic-pain	Naloxone + Morphine	89	36.9	USA	No	No
43	Eker et al.	2016	Double-blind, RCT	Knee, Arthritis, Chronic-pain	Lidocaine	52	65.15	Turkey	Yes	Yes
44	Kim et al.	2015	Double-blind, RCT	Cancer, Chronic-pain	Opioid	49	62	Korea	No	No
45	Kimos et al.	2007	Double-blind, P–C, RCT	Chronic-pain	Gabapentin	50	33.58	Canada	Yes	Yes
46	Narang et al.	2008	Double-blind, P–C, RCT, Crossover	Chronic-pain	Opioid	30	43.5	USA	No	No
47	Peyton et al.	2017	P–C, RCT	Postsurgical, Chronic-pain	Ketamine	80	55.3	Australia	No	No
48	Katz et al.	2005	P–C, RCT, Crossover	Low-back, Chronic-pain	Bupropion	60	49.8		Yes	Yes
49	Hashmi et al.	2012	Double-blind, P–C, RCT	Back, Chronic-pain	Lidocaine	30	51.36	USA	No	No
50	Shimoyama et al.	2014	Double-blind, P–C, RCT, Crossover	Cancer, Chronic-pain	Fentanyl	51	59.1	Japan	No	No
51	Wreje and Brorsson	1995	RCT	Chronic-pain	Sterile water	117	> = 25	Sweden	No	No
52	Han et al.	2016	Double-blind, P–C, RCT	Neuropathic, Chronic-pain	BTX-A	40	53.1	korea	Yes	Yes
53	Rauck et al.	2014	Double-blind, P–C, RCT	Chronic-pain	Hydrocodone	510	50.4	USA	No	No
54	Kim et al.	2010	Double-blind, P–C, RCT	Postsurgical, Chronic-pain	Pregabalin	94	39	Korea	No	No
55	Lee et al.	2019	RCT	Chronic-pain	BTX-A	60	50.9	Korea	No	No
56	Rashiq et al.	2003	Double-blind, P–C, RCT, Crossover	Low-back, Chronic-pain	Fentanyl	28	54		Yes	Yes
57	Kang et al.	2020	Double-blind, P–C, RCT	Postsurgical, Chronic-pain	Ketamine	168	50.8	korea	No	No
58	Lipton et al.	2021	P–C, RCT	Chronic-pain	Erenumab	955	41.1	Canada-13*	No	No
59	Williamson et al.	2014	P–C, RCT	Low-back, Knee, Arthritis, Chronic-pain	Duloxetine	780	63.2	Canada	No	No
60	Guo et al.	2020	RCT	Low-back, Chronic-pain	Celecoxib Eperisone	150	36	China	No	No
61	Damjanov et al.	2018	Double-blind, P–C, RCT	Chronic-pain	ACS	32	59		No	No
62	Abd-Elshafy et al.	2019	Double-blind, RCT	Postsurgical, Chronic-pain	Bupivacaine	60	35	Egypt	No	Yes
63	Levesque et al.	2021	Double-blind, RCT	Chronic-pain	BTX + Ropivacaïne	80	53.1		No	No
64	Maher et al.	2018	P–C, RCT	Chronic-pain	Ketamine	79	50.32	USA	No	No
65	Barry et al.	2019	RCT	Back, Chronic-pain	Methadone	40	37.7	USA	No	No
66	Shokeir and Mousa	2015	Double-blind, P–C, RCT	Chronic-pain	Bupivacaine	60	32.8	Egypt	Yes	Yes
67	Scudds et al.	1995	Double-blind, P–C, RCT	Chronic-pain	Lidocaine	61	46.1	Canada	No	No
68	Gimbel et al.	2016	Double-blind, P–C, RCT	Low-back, Chronic-pain	Buccal buprenorphine	510	52.8	USA	No	No
69	Matsuoka et al.	2019	P–C, RCT	Neuropathic, Cancer, Chronic-pain	Duloxetine	70	64.7	Japan	No	No
70	Yurekli et al.	2008	Double-blind, P–C, RCT	Chronic-pain	Sodium valproate	70	40	Turkey	Yes	Yes
71	Maarrawi et al.	2018	Double-blind, P–C, RCT	Chronic-pain	Amitriptyline	112	43.54	Lebanon	Yes	Yes
72	Li et al.	2018	Double-blind, RCT	Postsurgical, Chronic-pain	Ropivacaine + Dexamethasone	52	62	China	No	No
73	Almog et al.	2020	Double-blind, 3-arm, RCT, Crossover	Chronic-pain	THC	27	48.3	Israel	No	No
74	Wylde et al.	2015	Double-blind, RCT	Postsurgical, Knee, Chronic-pain	Bupivacaine	273	66	UK	No	No
75	Matsukawa et al.	2020	RCT	Chronic-pain	Cernitin + Tadalafil	100	65.9	Japan	No	No
76	Haddad et al.	2018	Double-blind, P–C, RCT, Crossover	Chronic-pain	Apomorphine	35	56.2	Israel	No	No
77	de Vries et al.	2016	Double-blind, P–C, RCT	Postsurgical, Chronic-pain	THC	65	52.2	Netherlands	Yes	Yes
78	Urquhart et al.	2018	Double-blind, RCT	Low-back, Chronic-pain	Amitriptyline	146	53.5	Australia	No	Yes
79	Lichtman et al.	2018	Double-blind, P–C, RCT	Cancer, Chronic-pain	Nabiximols	397	59.2	Belgium-12*	No	No
80	Schiphorst et al.	2014	Trible-Blind, P–C, RCT	Low-back, Chronic-pain	Acetaminophen/Tramadol	50	42	Netherlands	No	No
81	Cardenas et al.	2002	RCT	Chronic-pain	Amitriptyline	84	41	USA	Yes	Yes
82	Arnold et al.	2012	Double-blind, P–C, RCT	Chronic-pain	Milnacipran	1025	49.1	USA	No	No
83	Wasan et al.	2005	Double-blind, P–C, RCT, Crossover	Low-back, Chronic-pain	Morphine	20	44.2	USA	No	No
84	Baron et al.	2014	Double-blind, RCT	Neuropathic, Low-back, Chronic-pain	Tapentadol/Pregabalin	445	56.3	Germany	No	No
85	Portenoy et al.	2007	Double-blind, P–C, RCT	Low-back, Chronic-pain	Fentanyl	77	48.9	USA	No	No
86	Likar et al.	1997	Double-blind, RCT, Crossover	Arthritis, Chronic-pain	Morphine	21	68	Austria	No	No
87	Schwartzman et al.	2009	Double-blind, P–C, RCT	Chronic-pain	Ketamine	20	38	USA	Yes	Yes
88	Chu et al.	2012	Double-blind, P–C, RCT	Back, Chronic-pain	Morphine	139	44	USA	Yes	Yes
89	Sandrini et al.	2011	Double-blind, P–C, RCT	Chronic-pain	BoNTA	56	48.5	USA	No	No
90	Mahowald et al.	2009	Single-blinded, P–C, RCT	Arthritis, Chronic-pain	BoNTA	40	> = 48	USA	Yes	Yes
91	Loftus et al.	2010	Double-blind, P–C, RCT	Back, Chronic-pain	Ketamine	102	51.7	Lebanon /USA	Yes	Yes
92	Lehmann et al.	1997	P–C, RCT	Postsurgical, Chronic-pain	Fentanyl	29	44.15	USA	No	No
93	Kahlenberg et al.	2017	P–C, RCT	Chronic-pain	Celecoxib	98	34.2	USA	Yes	Yes
94	Silberstein et al.	2009	Double-blind, P–C, RCT	Chronic-pain	Topiramate	306	38.2	USA	No	No
95	Burgher et al.	2011	Double-blind, RCT	Low-back, Chronic-pain	Lidocaine	26	44.1	USA	No	No
96	McCleane	1999	Double-blind, P–C, RCT, Crossover	Neuropathic, Chronic-pain	Phenytoin	20	40	Ireland	Yes	Yes
97	Naliboff et al.	2011	2-arm, RCT	Chronic-pain	Opioid	135	52.7	USA	No	No
98	Booth et al.	2017	P–C, RCT	Postsurgical, Chronic-pain	Morphine	74	28	USA	No	Yes
99	Lee et al.	2006	Single-blinded, RCT	Chronic-pain	Rowatinex/Ibuprofen	50	44.2	Korea	No	No
100	Levendoglu et al.	2004	Double-blind, P–C, RCT, Crossover	Neuropathic, Chronic-pain	Gabapentin	20	35.9	Turkey	Yes	Yes
101	Yousef and Alzeftawy	2018	Double-blind, RCT	Chronic-pain	Opioid	100	53.44	Egypt	No	Yes
102	Yelland et al.	2009	Double-blind, P–C, RCT, Crossover	Neuropathic, Chronic-pain	Gabapentin	73	57.8	Australia	No	No
103	Yucel et al.	2004	Double-blind, P–C, RCT	Neuropathic, Chronic-pain	Venlafaxine	55	48.94	Turkey	No	No
104	Hudson et al.	2021	Double-blind, P–C, RCT	Knee, Arthritis, Chronic-pain	Nortriptyline	205	64.4	New Zealand	Yes	Yes
105	Rauck et al.	2006	Double-blind, P–C, RCT	Chronic-pain	Ziconotide	220	52.5	USA	No	No
106	Sandner-Kiesling et al.	2010	Double-blind	Noncancer, Chronic-pain	Naloxone + Oxycodone	379	56.2	Austria/Germany	No	No
107	Wang et al.	2017	RCT	Chronic-pain	Diosmin	300	41	China	No	Yes
108	Hawley et al.	2020	Double-blind, P–C, RCT, Crossover	Cancer, Chronic-pain	Lidocaine	25	53.76	Canada	No	No
109	Mathieson et al.	2017	Double-blind, P–C, RCT	Chronic-pain	Pregabalin	209	66	Australia	No	No
110	Wetzel et al.	2015	Double-blind, P–C, RCT, Crossover	Low-back, Noncancer, Chronic-pain	Nonopioid analgesic drugs	36	55	Austria	No	No
111	Khan et al.	2019	P–C, RCT	PostsurgicalNeuropathic, Cancer, Chronic-pain	Lidocaine + Pregabalin	100	55.2	Canada	No	No
112	Clarke et al.	112	Double-blind, RCT	Postsurgical, Chronic-pain	Gabapentin	126	58.9	Canada	Yes	Yes
113	Ma et al.	113	Double-blind, P–C, RCT	Chronic-pain	Oxycodone	116	58.2	China	Yes	Yes
114	J. H. Lee and C. S. Lee	114	Double-blind, P–C, RCT	Low-back, Chronic-pain	TA-ER	245	59.9	Korea	No	No
115	Imamura et al.	2016	Trible-Blind, RCT	Low-back, Chronic-pain	Lidocaine	378	48.26	Brazil	No	No
116	Baron et al.	2015	RCT	Neuropathic, Low-back, Chronic-pain	Tapentadol	258	58.1	Germany	No	No
117	Kim et al.	2017	Double-blind, RCT	Postsurgical, Cancer, Chronic-pain	Lidocaine + Magnesium	126	48.7	Korea	Yes	Yes
118	Iwamura et al.	2015	RCT	Chronic-pain	Eviprostat	100	50.1	Japan	No	No
119	Zhang et al.	2021	Double-blind, P–C, RCT	Chronic-pain	Ningmitai	120	33.7	China	No	No

Canada-13*: “Canada-13” was used as the group of 13 countries: “Canada, Austria, Belgium, Czech Republic, Finland, Germany, Poland, Slovakia, Sweden, the United Kingdom, Turkey, the Netherlands and USA”.

Belgium-12*: “Belgium-12” was used as the group of 12 countries: “Belgium, Bulgaria, Czechia, Germany, Hungary, Latvia, Lithuania, Poland, Puerto Rico, Romania, United Kingdom, United States”.

Opioids (Table 2) were tested in 32 (26.89%) studies with 5518 (31.16%) participants, where *Morphine*, *Oxycodone* and *Fentanyl* were common. *Lidocaine*, *Naloxone* and *Gabapentin* were the most frequently tested non-opioid drugs for chronic pain. The most common pain among chronic pain patients were lower back pain, which was explored in 26 (21.85%) studies with a pooled sample of 4626 (26.12%) while 13 studies reported chronic back pain among 1068 (6.03%) participants. The following pain types are post-surgical pain and neuropathic pain with 19 (15.97%) and 10 (8.4%) studies involved to test the efficiency of NSAID drugs on patients.

**Table 2:** Summary of drug and pain types included in systematic review.

Classes	Drug types	Studies (number, %)	Participants (number, %)
Opioids 32 (26.89%)	Oxycodone	4 (3.36%)	643 (3.63%)
	Fentanyl	4 (3.36%)	185 (1.04%)
	Methadone	1 (0.84%)	40 (0.23%)
	Morphine	9 (7.56%)	750 (4.24%)
	Buprenorphine	2 (1.68%)	659 (3.72%)
	Codeine	1 (0.84%)	58 (0.33%)
	Other Opioids	11 (9.24%)	3183 (17.97%)
Nonopioids	Naloxone	8 (6.72%)	1084 (6.12%)
	Gabapentin	8 (6.72%)	610 (3.44%)
	Lidocaine	10 (8.4%)	1036 (5.85%)
	Ketamine	5 (4.2%)	449 (2.54%)
	Amitriptyline	3 (2.52%)	342 (1.93%)
	Bupivacaine	4 (3.36%)	409 (2.31%)

Meta-analysis of mean difference of pain scores were applied to 24 studies with a sample of 2546 participants, producing a pooled mean difference (MD) of – 0.89 (95% CI [− 1·31, − 0·47]). There was a significant difference between chronic pain scores of patients taking NSAIDs compared to a placebo. Averagely, 0.89 point (0–10 scale) of pain reduction was observed based on the random effects model. A significant statistical drug efficiency was observed with BTX-A and Ketamine. A negative pooled mean difference was determined between BTX-A and Ketamine versus a placebo with a pain reduction of 0.98–1.26 based on a − 10 scale, respectively. Similar statistical results were not observed with other drugs in comparison to a placebo.

Within the common comparator as a “*placebo*”, the connected network included 34 studies, 52 pairwise comparisons, 32 interventions and 29 study designs. Gabapentin had a significant mean difference equalling to – 1.49 (95% CI [− 2⋅76, − 0⋅23], p-value < 0.05). Most interventions had a negative mean difference compared to a *placebo*, but a 95% CI covering 0 indicated insignificant effects for reducing pain. The results within the network were more conservative with the combination of direct and indirect evidence indicating most pharmaceutical interventions selected might have benefited from the “*placebo effect*”.

### Pairwise meta-analysis (PMA)

The PMA included 24 studies with pairwise comparisons between drugs and a placebo. The experimental and control group comprised of "Amitriptyline", "BTX-A”, “Gabapentin", "Ketamine", "Lidocaine", "Morphine", "Naloxone" and a placebo, respectively. A single study reported "Fentanyl", "Ningmitai", "THC", and "Oxycodone".

### PMA for baseline pain score

**Figure 2:**
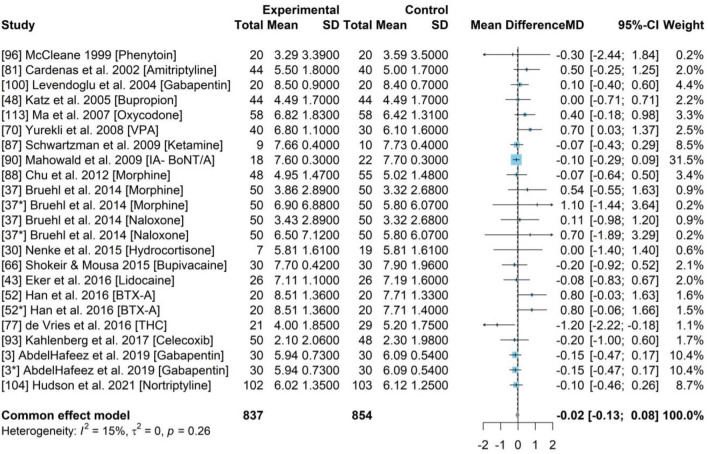
Forest plot for the baseline pain scores of experimental group and control group across 18 studies.

The PMA was used to test baseline pain score differences between the experimental and control group in 18 studies which comprised of a total sample of 1691 participants. The experimental and control groups comprised of 837 and 854 participants, respectively, with a pooled mean difference (MD) of – 0.02 (95% CI [− 0.13, 0.08]). The 95% CI was 0 and therefore, no statistically significant difference between baseline pain scores of two groups (Fig. 2). A weak statistical heterogeneity of 15% of ${I}^{2}$ (p = 0.26) was determined. This combined with the statistical insignificance indicates the randomisation of was completed accurately and that it is scientifically justifiable to use the post-treatment pain scores directly as the outcomes to evaluate treatment effects.

### PMA for drug efficacy between NSAID compared to a placebo

**Figure 3:**
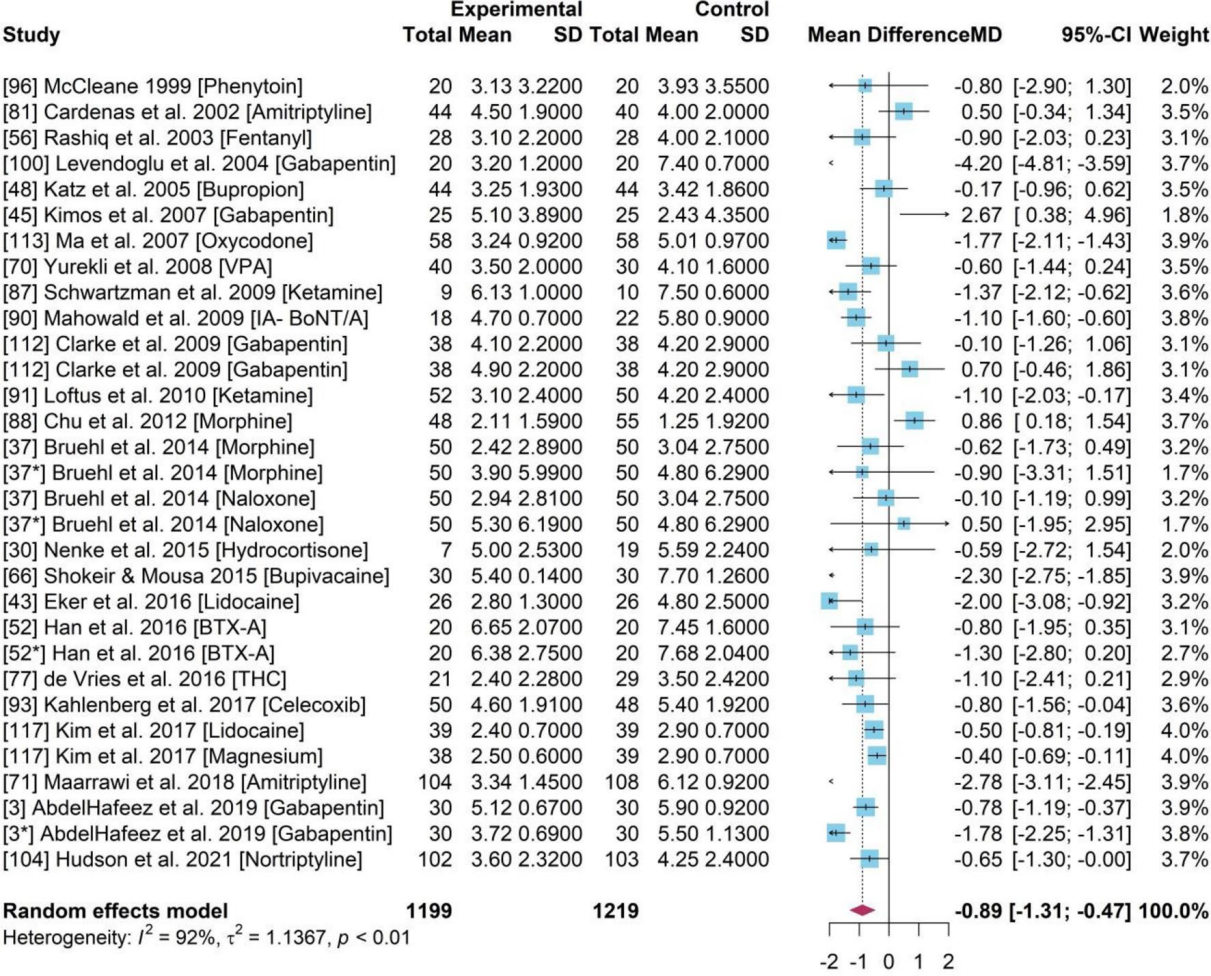
Forest plot for the pain scores of experimental group and control group across 24 studies testing all NSAID drugs (including some unnamed Opioids drugs).

This PMA included 24 studies (Fig. 3) with 2418 participants, with a MD of − 0.89 (95% CI [− 1.31, − 0.47]). The experimental and control group comprised of 1219 and 1199, respectively. A significant statistical heterogeneity of 92% of ${I}^{2}$ (p-value < 0.01) was identified. Mean difference (MD) was calculated to assess if there is statistically significant difference of post-treatment pain scores between experimental group and control group. The 95% CI was less than 0 which indicated a significant treatment effect with a reduction in pain by 0.89-point (0–10 scale) compared to those who were given a placebo.

### Meta-analyses

**Figure 4:**
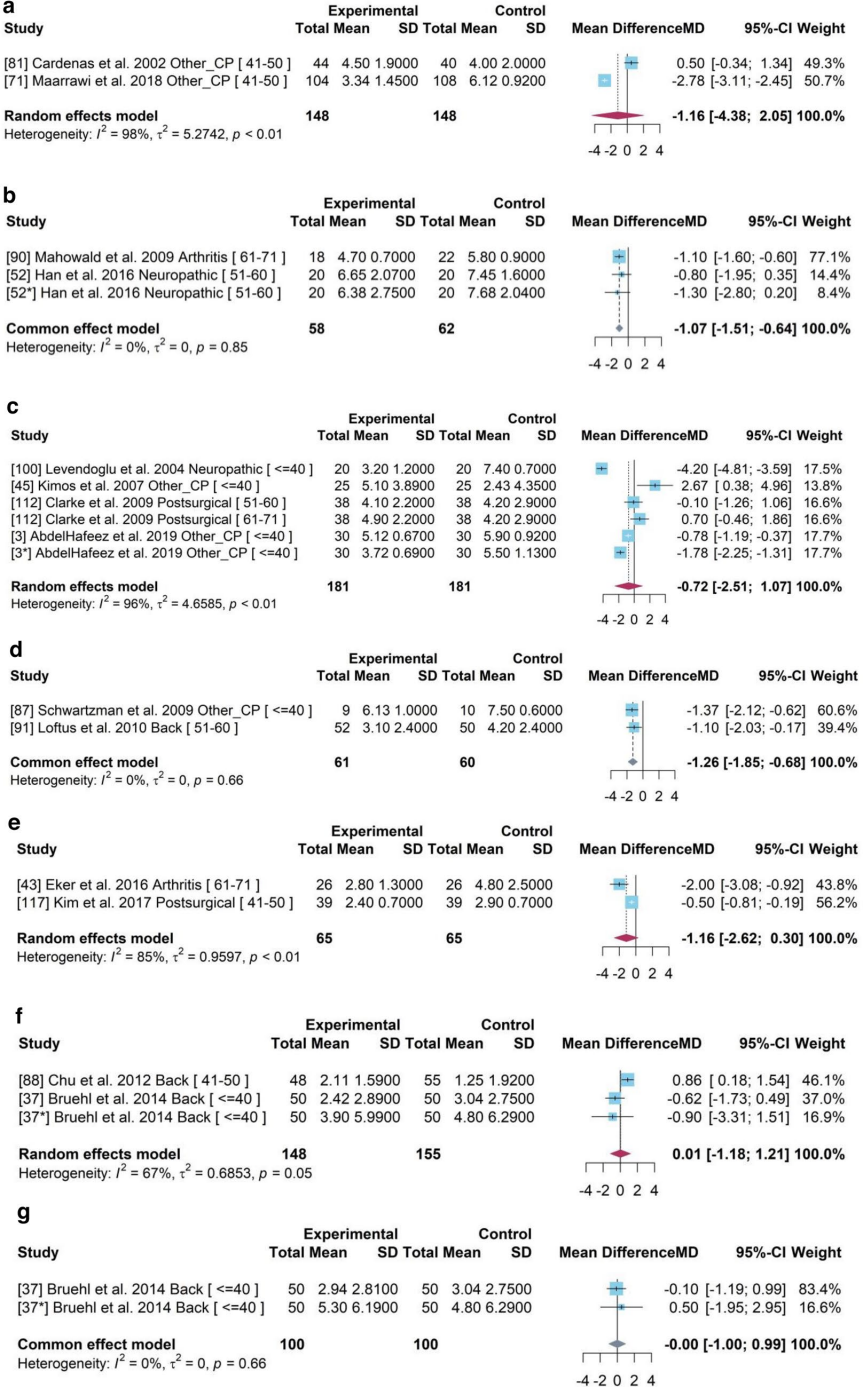
(**a**) Forest plot for drug efficiency of Amitriptyline. (**b**) Forest plot for drug efficiency of BTX-A. (**c**) Forest plot for drug efficiency of Gabapentin. (**d**) Forest plot for drug efficiency of Ketamine. (**e**) Forest plot for drug efficiency of Lidocaine. (**f**) Forest plot for drug efficiency of Morphine. (**g**) Forest plot for drug efficiency of Naloxone.

A statistically low heterogeneity of 0% of ${I}^{2}$ (p-value > 0.5) was identified among studies with *BTX-A, Ketamine* and *Naloxone* (Fig. 4b,d). *BTX-A* (Fig. 4b) and *Ketamine *(Fig. 4d) indicated statistically significant drug efficacy of – 1.07 [−1.51, − 0.64] and − 1.26 [− 1.85, − 0.68], respectively. The treatment efficiency compared to the placebo had a 1 point pain reduction within a 0–10 evaluation scale. Ketamine demonstrated optimal efficacy with a 1·26 point pain reduction on average.

The PMA for *BTX-A* (Fig. 4b) and *Naloxone* (Fig. 4g) showed a low heterogeneity as the data was pooled from a single study.

Studies on *Amitriptyline, Gabapentin, Lidocaine and Morphine* had a high heterogeneity and a statistically insignificant drug efficacy (Fig. 4a,c,e,f). The mean difference of 95% CI was 0 indicating an insignificant treatment difference between the drugs and placebo based on the random effects model.

### Opioids drugs

**Figure 5:**
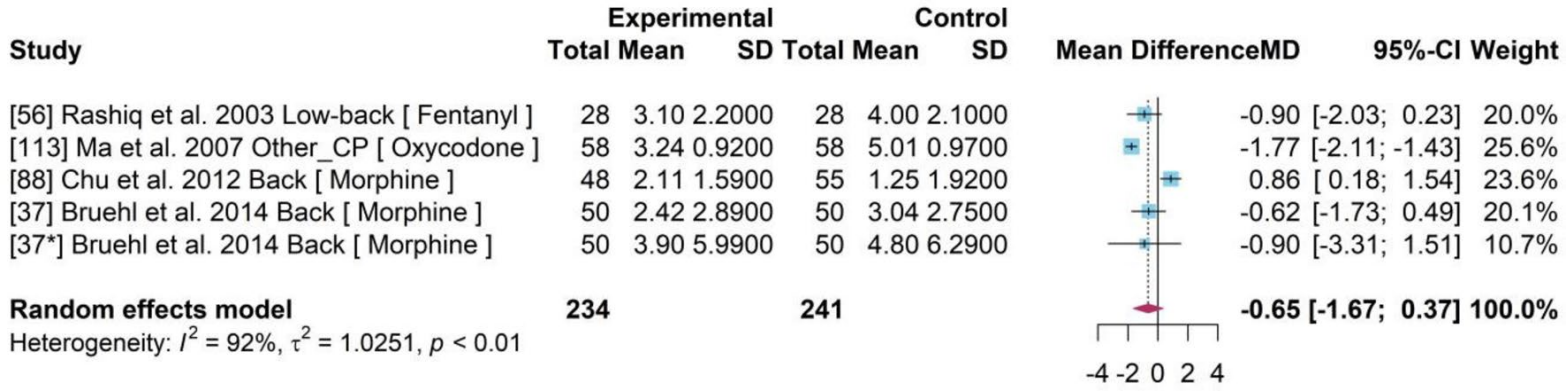
Forest plot for drug efficiency of Opioids drugs*.

A meta-analysis was conducted with 4 studies (Fig. 5). A pooled MD of – 0.65 and a 95% CI [− 1.67, 0.37] was determined indicating an insignificant treatment effect of opioids drugs compared to a placebo. A statistically significant heterogeneity of 92% of ${I}^{2}$ (p-value < 0·01) was identified.

### Network meta-analysis (NMA)

**Figure 6:**
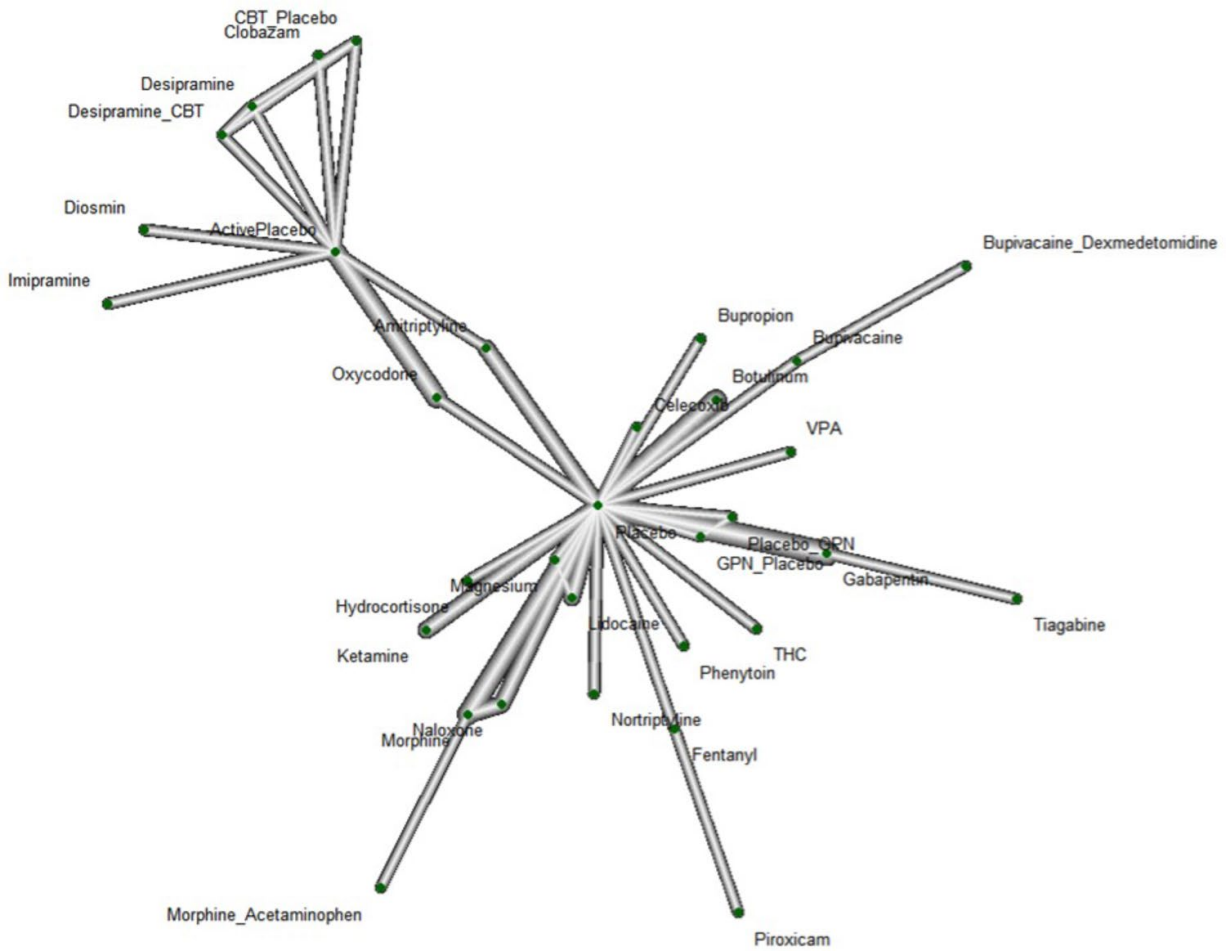
Network plot where Placebo was the reference group with 34 studies and 32 interventions.

A NMA (Fig. 6) was completed for 34 studies. The nodes correspond to each intervention included within the network where the interventions with direct comparisons are linked with a line. The thickness of lines corresponds to the number of trials evaluating the comparison. A connected network was built based on the *placebo* which was mostly *Tolterodine* based on the original studies. The evaluations between interventions were supported by direct comparison and indirect comparison.

**Figure 7:**
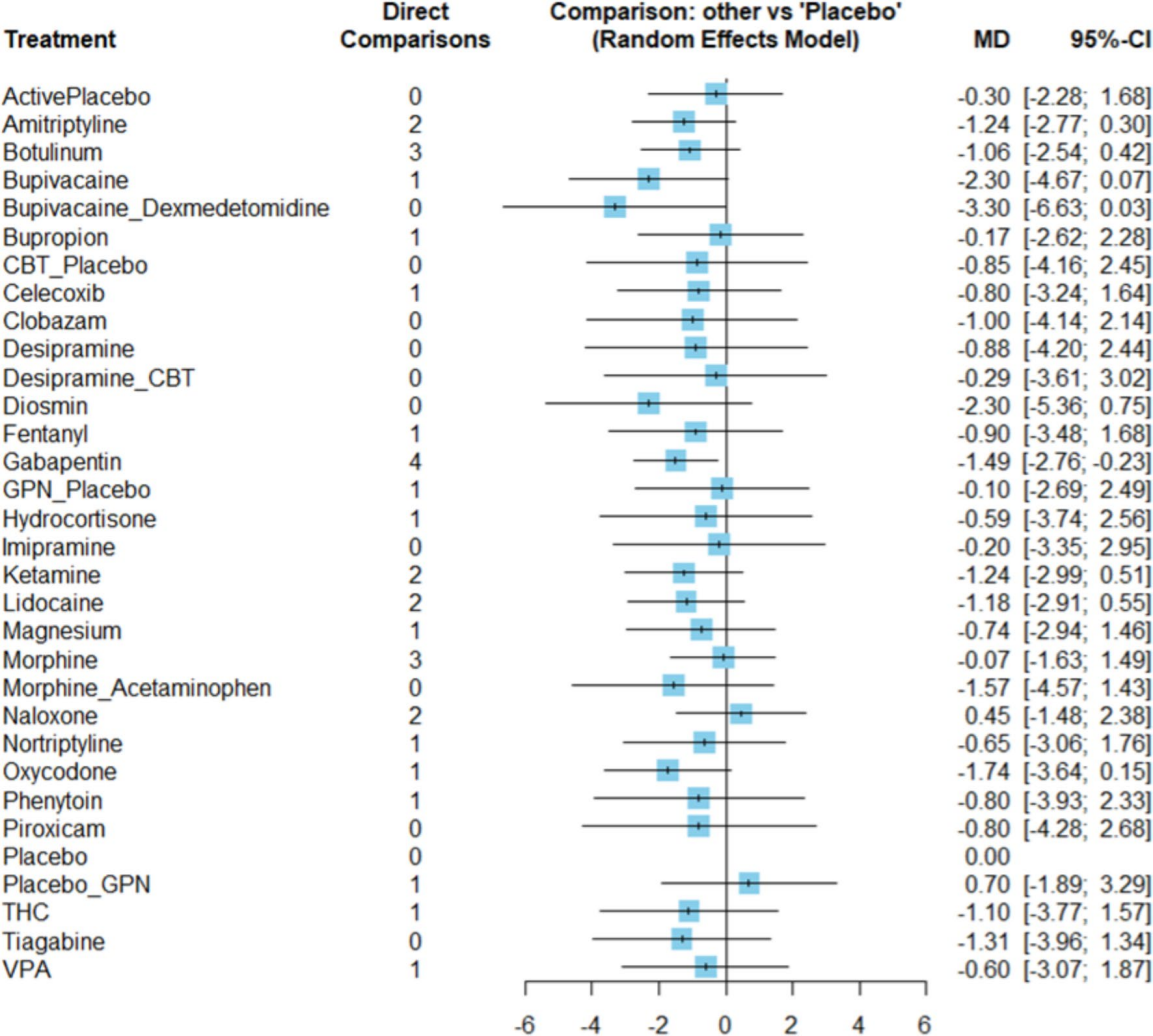
Forest plot for intervention efficiency compared to Placebo in NMA.

**Figure 8:**
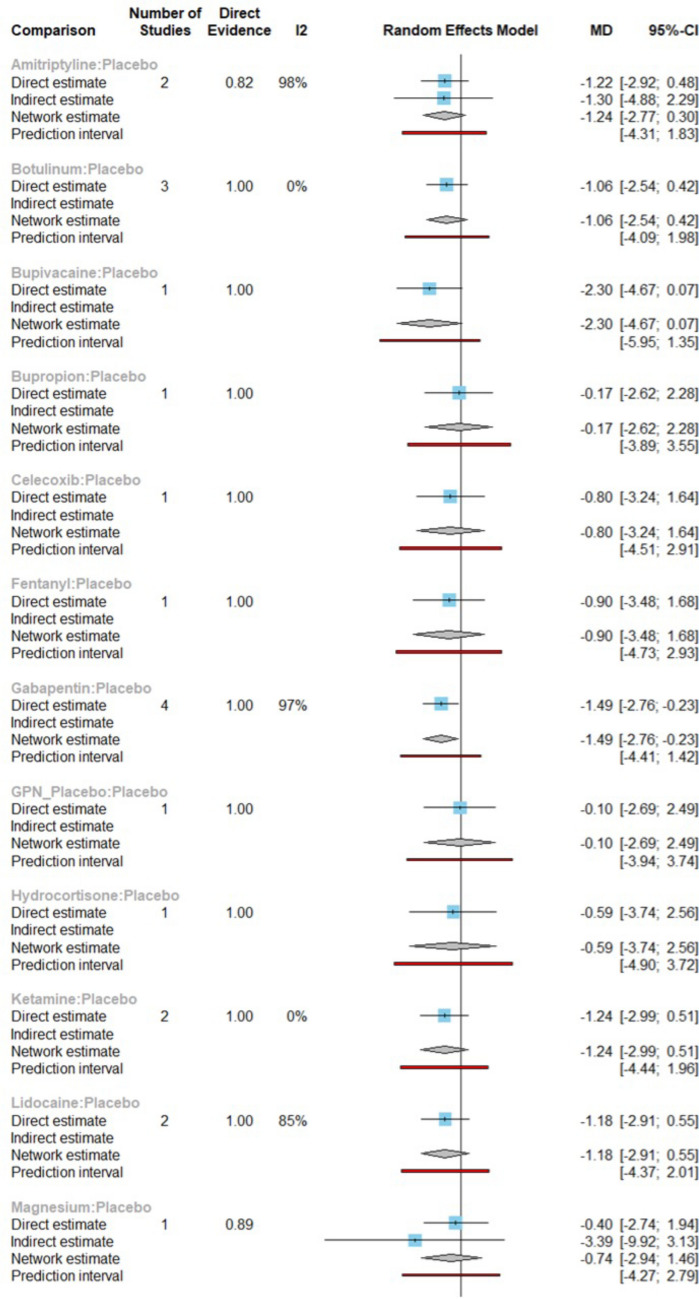
Forest plot for intervention efficiency compared to Placebo in NMA with detailed direct and indirect comparisons.

In the network with the placebo as the reference group, *Gabapentin* (Fig. 7) comprised of a MD equaling to – 1.49 (95% CI [− 2.76, − 0.23], p-value < 0.05) indicating a significant effect on reducing chronic pain and direct comparisons were made using 4 studies (Fig. 8a). The pooled MD of *Botulinum* and *Ketamine* were −1.06 and – 1.24, respectively. These were similar to the results in the PWA, but their 95% CI was 0 therefore showed insignificant effect on pain reduction compared to a placebo. Most combined interventions had a negative MD compared to a placebo with a 95% CI of 0 indicated statistically insignificant results for reducing pain.

*Imipramine*, *Diosimin*, *Desipramine*, *Clobazam*, *Piroxicam* and *Tiagabine* had not been directly compared to a placebo based on the identified data therefore the comparative treatment effected between them and a placebo was not possible to complete.

### Subgroup analysis

**Figure 9:**
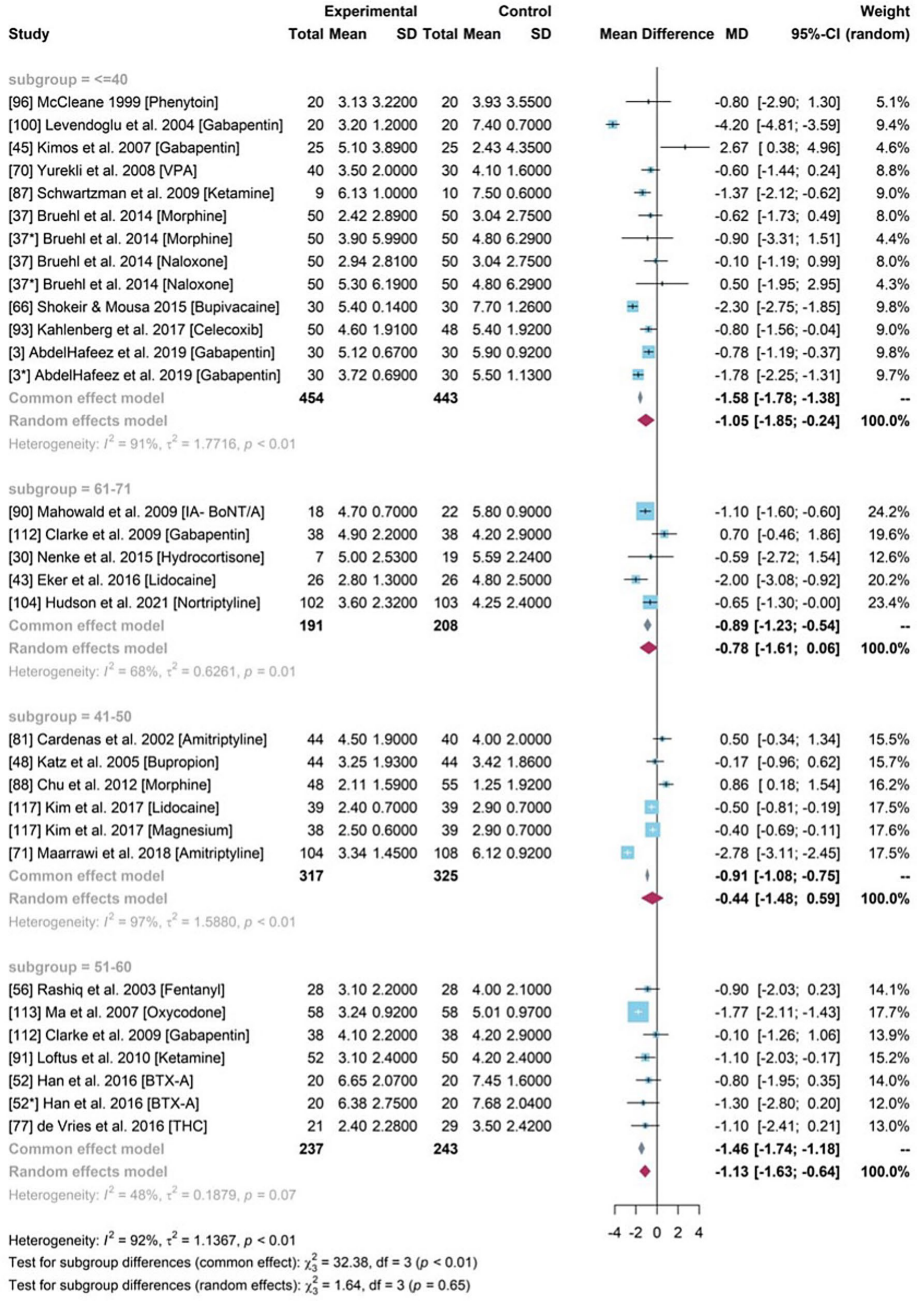
Forest plot for the mean difference of pain scores between experimental group and control group across different mean age of participants.

A subgroup analyses was conducted for 24 studies within the meta-analysis to explore the sources of heterogeneity and unbiased estimation based on age, pain type, period and geographical location (Fig. 9). The sub-group analysis for pain type, time period and geographical location can be found in the Supplementary file whilst average age is shown below.

### Subgroup analysis for pain core difference based on different age groups

It showed that the heterogeneity among studies with participants who were older than 50 years old had changed with decreased *I*^2^ (*I*^2^ = 48% for “51–60”, *I*^2^ = 68% for “61–71”). A common effects model was chosen for subgroup “51–60”, which produced a higher estimation of pain reduction with a mean difference of – 1.46 (95% CI [− 1.74, − 1.18]). Based on the high heterogeneity (*I*^2^ > 50%), random effects models were built for other subgroups. The group with participants younger than 40 years older obtained a significant drug efficiency (MD − 1·05, 95% CI [− 1.85, − 0.24]). The pooled drug effects (Fig. 9) in the 41–50 and 61–71 years of age groups were much lower than the overall treatment effect of NSAID drugs identified in the PMA. The 95% CI of 0 indicated statistically ineffective compared to the placebo. The random effects models showed the decrease of heterogeneity indicating that age may be a source of heterogeneity.

### Sensitivity analysis

**Figure 10:**
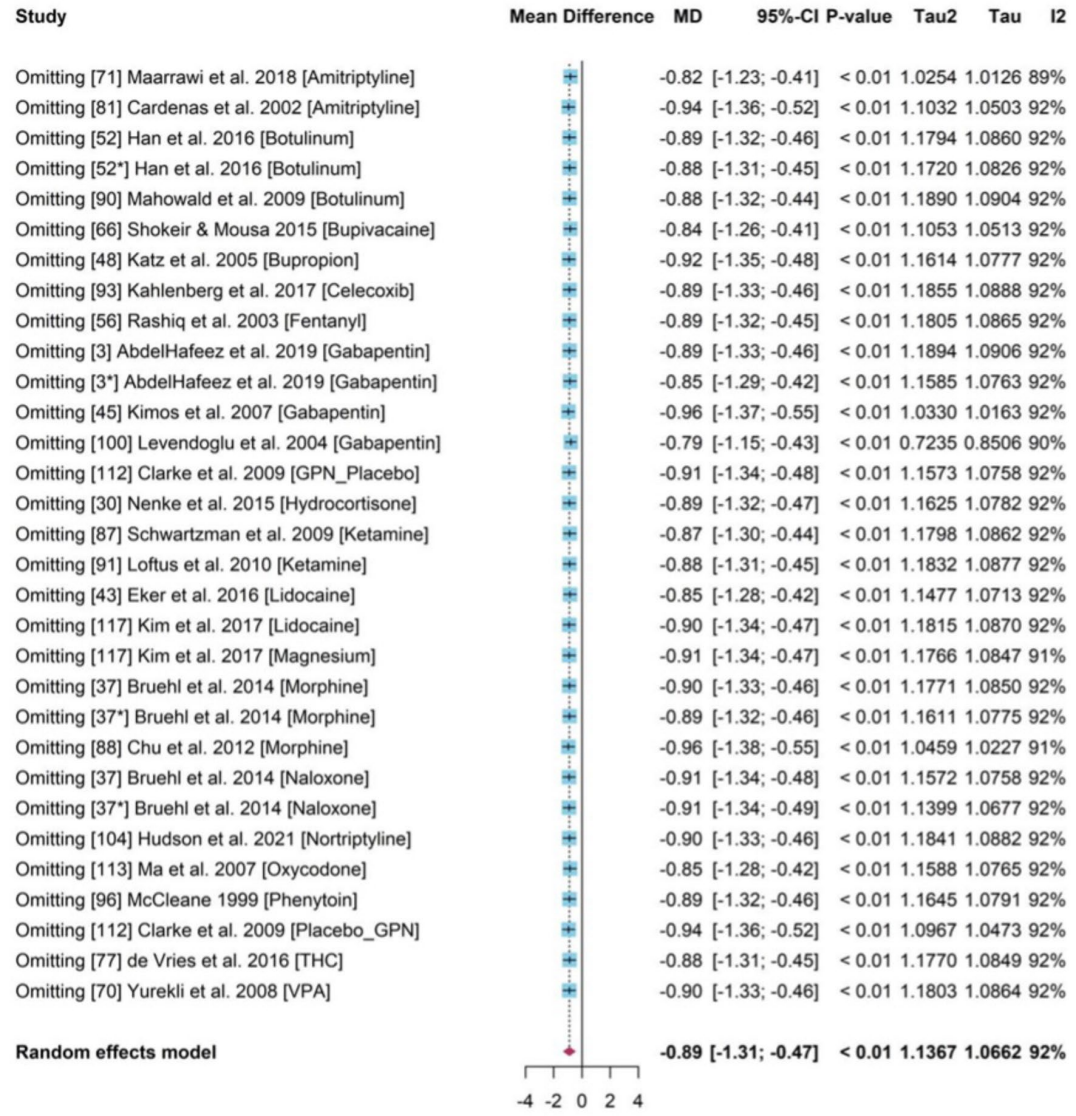
Forest plot for sensitivity analysis with studies in MA.

The sensitivity analysis was conducted (Fig. 10) for the PMA where some studies influenced the pooled results compared to the overall estimation (− 0.89). To test this theory, study number 71 and 100 were omitted and the pooled results were much lower, − 0.82 and – 0.79, respectively. Studies with *Amitriptyline* and *Gabapentin* produced unstable treatment results, and the absence of these showed an overestimation (study 81, 45) or underestimates (study 71, 100). Collectively, the high heterogeneity (*I*^2^ = 92% p-value < 0.01) was stable and a robust treatment effect with negative mean differences and a significant 95% CI remained. Therefore, the pooled treatment effects identified was credible.

### Publication bias

**Figure 11:**
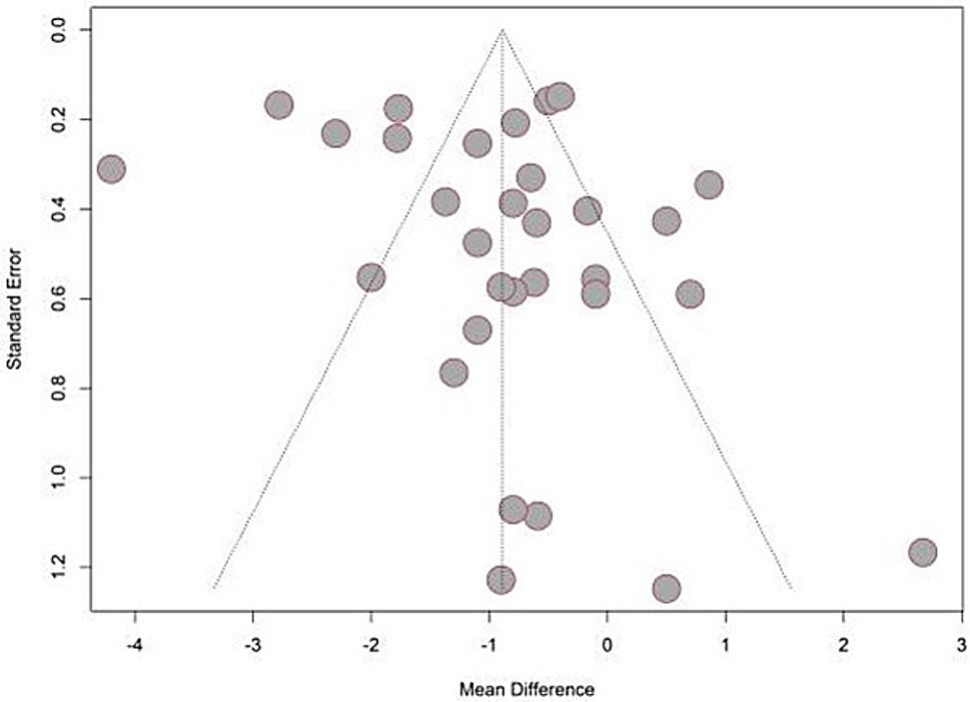
Funnel plot for studies used in PMA.

The funnel plots (Fig. 11) within the PMA indicated symmetry. Although several studies were not within the remit of the funnel, the Egger’s test showed a p value (0.22) larger than 0.05 which indicated the lack of small-study effects (Table 3).

**Table 3:** Egger test results for studies used in PMA.

Test result	t = 1.24, df = 29, p-value = 0.2247			
Sample estimates	Bias	SE.bias	Intercept	SE.intercept
	1.49	1.2	− 1.593	0.3737

## Discussion

We identified opioids and non-opioids were the two primary classes of pharmacological interventions in chronic pain management. Opioids are widely used in the management of cancer pain and non-cancer associated pain^20,21^. The long-term use of opioids in the management of chronic non-malignant pain has come under scrutiny more recently and is now recommended only if benefits of initiating treatment would significantly outweigh the potential risks, and possibly as an adjunct to the primary intervention^22,23^. Our study has shown that judicious use of non-opioid medications along with other treatment modalities could provide better outcomes in managing chronic pain thereby removing long-term side-effects observed during opioid therapy. With cancer patients increasingly being cured or achieving long term remission, prolonged use of opioids could result in aberrant behaviour and dependence. Awareness of an opioid crisis globally has prompted clinicians to exercise caution in their prescription habits, but the WHO supports the use of opioids including Fentanyl and Methadone as an essential class of medication for the management of cancer pain^24,25^.

The meta-analysis of baseline pain scores lacked statistical significance between experimental and control groups. The significant reduction in chronic pain scores of patients taking NSAID versus non-steroidal opioid drugs compared to patients given placebo under a random effects model. The presence of a significant drug efficiency with *BTX-A* and *Ketamine* is interesting although the pooled results of other drugs and interventions had statistically insignificant results with a 95% CI of 0. The pooled evidence indicated Ketamine showed the highest pain reduction (1.26) followed by BTX-A (0.98). Studies testing on other drugs including Amitriptyline, Gabapentin, Morphine and Lidocaine had a high heterogeneity and insignificant drug efficiency. Overall, evidence from the PMA showed a strong efficacy within the NSAIDs group with managing pain which were remarkably narrowed when exclusive trials with low risk of bias were included^26–28^.

In this study, a pairwise meta-analysis and NMA consolidating the evidence of 46 studies was carried out, with the former comparing several different opioids. Morphine has traditionally been used for the management of moderate to severe chronic pain^29^. Despite morphine being a potent analgesic [MD 0.01 (95% CI [− 1.18, 1.21], newer opioids are now being employed owing to their superior safety profile. Oxycodone and Fentanyl appear to be popular due to better availability and vast clinical experience including the well accepted effectiveness demonstrated, as per patient and clinically reported outcomes. Our results are aligned to these trends where the effectiveness is shown to include a MD 1.77 (95% CI [− 2.11, − 1.43]) for Oxycodone and a MD of − 0.90 (95% CI [− 2.03, 0.23])] for Fentanyl (32). However, untoward gastrointestinal effects (constipation, nausea, and vomiting) still remain a major concern with opioid use and are often responsible for discontinuation of treatment^30,31^. Recent evidence favours the use of a combination of oxycodone and naloxone in patients with chronic pain (after ensuring that there is no cause for porto-systemic anastomosis), to offer an improved bowel function without any effective change in analgesia^32^. The concerns of developing tolerance, opioid-induced hyperalgesia, aberrant behaviour and dependence with opioids is a pragmatic reason to develop effective alternative treatment modalities especially for vulnerable individuals. In pairwise comparison, we observed Ketamine to be superior to other pharmacological interventions with a mean difference MD − 1.26 (95% CI [− 1.85, − 0.68]).

There are several guidelines recommending the use of Pregabalin, Gabapentin, Duloxetine, and Amitriptyline as first line drugs in the management of neuropathic pain^33–35^. However, the use of gabapentinoids is being challenged as it lacks favourable robust evidence for efficacy against pain syndromes other than fibromyalgia, post herpetic neuralgia and diabetic neuropathy, and many clinicians have also highlighted the potential for misuse and developing dependence^36–38^. The use of BTX-A, Ketamine, Ningmitai and THC for the management of various chronic pain conditions is popular and well established^39–43^ and our study shows the effective use of these as analgesics when compared to placebo. There is evidence to support the efficacy of BTX-A for the management of neuropathic pain although the sample sizes used in the studies were small and therefore the real-world applicability remains limited^29^. BTX-A is also used in management of myofascial pains^44,45^ although further evidence on the efficacy and tolerability within all populations, especially those with existing co-morbidities needs to be evaluated. Ketamine was found to be beneficial in managing some neuropathic pains^46^ and as an infusion the rates of serious adverse effects were found to be similar to placebo^47,48^. Further studies are required to gather evidence to better understand its psychedelic effects and its role in the management of PTSD, anxiety and depression. A renewed use of magnesium in managing chronic pain has been demonstrated in some literature^49^. Our results indicate similar evidence in the use of magnesium, but will require further research to determine the efficacy, safety and effectiveness in managing short, medium and long-term pain.

The NMA provided more reliable results with direct and indirect comparisons between different drugs under different study designs. However, only a small number of multi-arm trials were eligible and the distribution of trials studying different drugs was uneven. It resulted in the lack of direct evidence of certain drugs and their relative efficacy in the network was unstable due to excessive reliance on indirect comparisons. Therefore, well designed and robust clinical trials should be conducted to verify the efficacy of pharmaceutical interventions used in chronic pain management.

## Conclusion

To the best of our knowledge, this is the first pairwise MA and NMA reporting the synthesis of the prevalence of the efficacy of pharmacological treatments used in the management of chronic pain with a large sample size of 17,708 participants. Management of long-term chronic pain needs to be prioritised for several reasons including humanitarian, the strain on the healthcare systems and the impact on the economy due to loss of productivity. The use of pharmaceutical agents in the long-term management of chronic pain has been debated for several decades, yet there has not been a consensus on this matter. This study supports the importance of generating better evidence by way of robust clinical trials, the need for drafting clinical guidelines that is pragmatic, practical as well as clinically significant and the use of better data-connectivity methods to improve clinical practice in the real-world.

### Supplementary Information

Supplementary Information 1.

## Data Availability

The authors will consider sharing the dataset gathered upon receipt of reasonable requests.

## Appendix

See Table 4.

**Table 4:** Interventions used in studies.

Study number	Author	Intervention abbreviation	Intervention details
1	Weizman et al.	THC	Cannabis
2	Krebs et al.	Opioid	Opioid and nonopioid medication therapy
3	AbdelHafeez et al.	Gabapentin	Gabapentin 2700 mg daily
4	Bushey et al.	Opioid	Analgesic
5	Bruehl et al.	Morphine + Naloxone	Morphine and Naloxone
6	Worley et al.	Buprenorphine/Naloxone	Buprenorphine/naloxone
7	Dindo et al.	ACT	Acceptance and Commitment Therapy
8	Hruschak et al.	IPGT	Psychoeducation, motivational interviewing, cognitive Behavioral therapy, mindfulness, and peer suppor
9	Azevedo et al.	Opioid	Opioid
10	Gudin et al.	NKTR-181	NKTR-181 administered at doses of 100–600 mg twice daily
11	Stahl et al.	Venlafaxine	Lower-dose venlafaxine (≤ 150 mg/day)
12	Schliessbach et al.	Imipramine	Imipramine 75
13	Mohamed et al.	Morphine	Topical morphine (in 1 of 3 doses: 5, 10, or 15 mg)
14	Schliessbach et al.	Oxycodone + Imipramine + Clobazam	Oxycodone 15 mg, imipramine 75 mg, clobazam 20 mg
15	Hermans et al.	Naloxone	0 mg morphine or 0.2 mg/mL Naloxone) and placebo (2 mL Aqua) group
16	Todorov et al.	Gabapentin + Tiagabine	Gabapentin and Tiagabine
17	Sadatsune et al.	Gabapentin	Gabapentin Group received 600 mg of gabapentin preoperatively, one hour prior to surgery, and Control Group received placebo
18	Edwards et al.	Opioid	Oral opioid therapy
19	Katz et al.	Naproxen + Tanezumab	Intravenous tanezumab 200 μg/kg plus oral placebo (n = 88), intravenous placebo plus oral naproxen 500 mg twice a day (n = 88), or intravenous placebo plus oral placebo (n = 41)
20	Hayek et al.	Opioid + Bupivacaine	opioid with bupivacaine
21	Schliessbach et al.	Clobazam	Received a single oral dose of clobazam 20 mg or active placebo tolterodine 1 mg
22	Bruehl et al.	Opioid	Opioid
23	Kim et al.	Nefopam	Infused with the same volume of saline or nefopam (0.2 mg/kg bolus, 120 μg/kg/h continuous infusion) during surgery
24	Eisenach et al.	Ketorolac	Drug administration
25	Rauck et al.	Adenosine/Clonidine	Intrathecal clonidine, 100 μg, or adenosine, 2 mg
26	Buchheit et al.	Valproate	Oral valproic acid
27	Papadokostakis et al.	Calcitonin	200 IU intranasal salmon calcitonin and 1000 mg of oral calcium daily
28	Gould et al.	Desipramine	Desipramine titrated to reach a serum concentration level of 15 to 65 ng/mL;
29	Schnitzer et al.	D-cycloserine	d-Cycloserine
30	Nenke et al.	Hydrocortisone	10 mg/m^2^/day of oral hydrocortisone in three divided doses o
31	Sopata et al.	Opioid	opioids
32	Kendall et al.	Lidocaine	1.5 mg/kg bolus of intravenous lidocaine followed by a 2 mg/kg/h infusion
33	Hongo et al.	Risedronate + Elcatonin	risedronate plus elcatonin
34	Amr and Yousef	Venlafaxine + Gabapentin	Venlafaxine 37.5 mg/day, gabapentin 300 mg/day
35	Pedersen et al.	Codeine + Paracetamol	30 mg codeine and 400 or 500 mg of paracetamol
36	Choi et al.	Lidocaine	The patients received 2 mg/kg of lidocaine followed by continuous infusions of 3 mg/kg/h of lidocaine
37	Bruehl et al.	Morphine + Naloxone	Naloxone (8 mg), morphine (0.08 mg/kg)
38	Chrubasik et al.	Capsicum	Cream containing capsaicin 0.05%
39	Schliessbach et al.	Oxycodone	Oxycodone 15 mg
40	Bruehl and Chung	Naloxone	8 mg dose of naloxone
41	Bruehl et al.	Naloxone + Morphine	Naloxone, morphine
42	Burns et al.	Naloxone + Morphine	Naloxone and morphine
43	Eker et al.	Lidocaine	Group I (n = 26) received 7 mL 0.5% lidocaine
44	Kim et al.	Opioid	Opioid therapy
45	Kimos et al.	Gabapentin	Gabapentin
46	Narang et al.	Opioid	Opioids
47	Peyton et al.	Ketamine	Ketamine
48	Katz et al.	Bupropion	Bupropion
49	Hashmi et al.	Lidocaine	Lidocaine
50	Shimoyama et al.	Fentanyl	Fentanyl
51	Wreje and Brorsson	Sterile water	Sterile water
52	Han et al.	BTX-A	Botulinum toxin type A
53	Rauck et al.	Hydrocodone	Hydrocodone 20–100 mg every 12 h)
54	Kim et al.	Pregabalin	pregabalin
55	Lee et al.	BTX-A	Botulinum Toxin Injection
56	Rashiq et al.	Fentanyl	Opioid
57	Kang et al.	Ketamine	0.12 mg/kg/h of ketamine
58	Lipton et al.	Erenumab	Erenumab 70 and 140 mg
59	Williamson et al.	Duloxetine	Duloxetine
60	Guo et al.	Celecoxib Eperisone	Celecoxib Eperisone
61	Damjanov et al.	ACS	Autologous conditioned serum (ACS; marketed as Orthokine®)
62	Abd-Elshafy et al.	Bupivacaine	Drug: Dexmedetomidine isobaric bupivacaine 0.5% (0.3 ml/kg) and dexmedetomidine (1 mcg/kg) Drug: Bupivacaine isobaric bupivacaine 0.5% (0.3 ml/kg)
63	Levesque et al.	BTX + Ropivacaïne	Drug: botulinum toxin A + ropivacaïne Drug: Ropivacaïne
64	Maher et al.	Ketamine	Ketamine
65	Barry et al.	Methadone	Methadone
66	Shokeir and Mousa	Bupivacaine	Bupivacaine
67	Scudds et al.	Lidocaine	Lidocaine
68	Gimbel et al.	Buccal buprenorphine	Buccal buprenorphine
69	Matsuoka et al.	Duloxetine	Duloxetine 20 mg
70	Yurekli et al.	Sodium valproate	Sodium valproate
71	Maarrawi et al.	Amitriptyline	Amitriptyline
72	Li et al.	Ropivacaine + Dexamethasone	Single 20-ml injection of 0.50% ropivacaine plus 10 mg dexamethasone
73	Almog et al.	THC	THC: 0.5 mg, 1 mg
74	Wylde et al.	Bupivacaine	Anaesthetic with 3 mL of 0.5% plain bupivacaine
75	Matsukawa et al.	Cernitin + Tadalafil	Tadalafil
76	Haddad et al.	Apomorphine	Apomorphine
77	de Vries et al.	THC	Tetrahydrocannabinol
78	Urquhart et al.	Amitriptyline	Amitriptyline; 25 mg per day
79	Lichtman et al.	Nabiximols	Nabiximols
80	Schiphorst et al.	Acetaminophen/Tramadol	Acetaminophen/tramadol 325 mg/37.5 mg
81	Cardenas et al.	Amitriptyline	Amitriptyline
82	Arnold et al.	Milnacipran	Chronic pain
83	Wasan et al.	Morphine	Morphine
84	Baron et al.	Tapentadol/Pregabalin	Tapentadol PR 300 mg/day + pregabalin
85	Portenoy et al.	Fentanyl	Fentanyl
86	Likar et al.	Morphine	Morphine hydrochloride
87	Schwartzman et al.	Ketamine	Ketamine
88	Chu et al.	Morphine	Morphine
89	Sandrini et al.	BoNTA	Onabotulinum toxin A
90	Mahowald et al.	BoNTA	Botulinum Toxin Type A
91	Loftus et al.	Ketamine	Ketamine infusions
92	Lehmann et al.	Fentanyl	Transdermal fentanyl
93	Kahlenberg et al.	Celecoxib	Celecoxib
94	Silberstein et al.	Topiramate	Topiramate
95	Burgher et al.	Lidocaine	Lidocaine and either clonidine (200 or 400mcg) or triamcinolone
96	McCleane	Phenytoin	Phenytoin (Parke Davis)
97	Naliboff et al.	Opioid	Opioids
98	Booth et al.	Morphine	300 mcg spinal morphine and 1 g acetaminophen
99	Lee et al.	Rowatinex/Ibuprofen	Rowatinex 200 mg/ibuprofen 600 mg
100	Levendoglu et al.	Gabapentin	Gabapentin
101	Yousef and Alzeftawy	Opioid	Oral perixicam
102	Yelland et al.	Gabapentin	Gabapentin
103	Yucel et al.	Venlafaxine	Venlafaxine
104	Hudson et al.	Nortriptyline	Nortriptyline
105	Rauck et al.	Ziconotide	Ziconotide
106	Sandner-Kiesling et al.	Naloxone + Oxycodone	Oxycodone PR/naloxone PR
107	Wang et al.	Diosmin	Diosmin
108	Hawley et al.	Lidocaine	Lidocaine
109	Mathieson et al.	Pregabalin	Pregabalin at a dose of 150 mg
110	Wetzel et al.	Nonopioid analgesic drugs	Oral nonopioid analgesic drug
111	Khan et al.	Lidocaine + Pregabalin	Pregabalin
112	Clarke et al.	Gabapentin	Gabapentin
113	Ma et al.	Oxycodone	Oxycodone
114	J. H. Lee and C. S. Lee	TA-ER	Tramadol hydrochloride 75-mg/acetaminophen 650-mg
115	Imamura et al.	Lidocaine	Paraspinous lidocaine injection
116	Baron et al.	Tapentadol	Tapentadol
117	Kim et al.	Lidocaine + Magnesium	Lidocaine (L), magnesium (M
118	Iwamura et al.	Eviprostat	Eviprostat
119	Zhang et al.	Ningmitai	Ningmitai Capsule

## Code availability

The authors will consider sharing the novel code created upon receipt of reasonable requests.
