# Supplementary material for: A systematic review and network meta-analysis of pharmaceutical interventions used to manage chronic pain
Source: Sci Rep. 2024 Jan 18;14:1621. doi: 10.1038/s41598-023-49761-3 (PMC10796361; doi:10.1038/s41598-023-49761-3)
Supplement: Supplementary file 1 — Supplementary Information 1. [file 41598_2023_49761_MOESM1_ESM.docx]

**Statistical analysis plan**

A pairwise meta-analysis (PMA) is a statistical combination of the results of two or more independent studies comparing only two interventions. When the comparative effects of a range of interventions are concerned, appropriate statistical methods must be used for analysis. Network meta-analysis (NMA) is a natural extension of classical PMA and can be used to compare all treatments that are connected in a network ^13^. The fundamental difference between them is that PMA produces only one estimate of pooling effects from the selected pair of interventions, while NMA produces multiple comparative estimates of pooling effects by connecting all alternative interventions ^14^.

NMA is preferable over conventional PMA by applying indirect evidence to justify comparisons amongst all treatments with more precise estimation. By integrating both direct and indirect effects from the entire set of evidence, NMA provides a global estimate of the efficacy or safety of numerous experimental treatments ^15^ .NMA formalizes this process in a statistical model by combining results from all the studies simultaneously, which enables us to obtain relative treatment effects for all pairs of interventions even for those without direct evidence.

NMA allows for the comparison of two or more interventions of interest that have never been directly compared provided that studies examining these treatments were linked by a common comparator ^19^. By incorporating direct and indirect treatment comparisons NMA provided greater statistical precision compared to PMA ^20^. Rankings of a set of drugs or combined interventions for relieving chronic pain with respect to their clinically efficacy can be calculated based on the Network models. Homogeneity and Consistency were tested to see if the assumptions in NMA were violated.

***Network Meta-Analysis***

Treatment effects (TE) and standard errors of treatment effects (seTE) were calculated by the following equations, where Mean value, Standard deviation (SD) and Sample size (N) were used (1 for experimental group, 2 for control group).

(a) TE = Mean − Mean 12

(b) seTE = SD12 + SD22 N1 N2

*Subgroup analysis for pain score difference based on different time periods*

A high heterogeneity of 94% and 87% of $I^{2}$ (p-value < 0.01) was identified in studies with study periods lasting for months and weeks, respectively (Figure 10). It can be explained that the long follow-up time wass common in RCTs and different studies might be heterogeneous by involving more different covariates, assessment tools and other factors. However, as the studies with testing periods of minutes and hours were limited, the decrease of *I^2^* was not supportive for showing the source of heterogeneity. After grouping based on testing intervals, studies for Months had a better treatment effect with a mean difference of -1.05 (95% CI = [-1.77, -0.33]) compared to Placebo. But the group of Weeks (-0.83) and group of Hours (-0.47) had significant results but decreased absolute values of pain reduction. An insignificant pain reduction in group of Minutes with 95% CI covering 0 (MD = -0.47, 95% CI = [-1.09, 0.11]). Due to the unbalanced study distribution (study numbers and designs), it was not convincing to treat the testing period as the source of differences in heterogeneity and drug efficacy.

*Subgroup analysis for pain score difference based on different pain types*

Based on the Pain Types of chronic pain patients in studies, 6 subgroups were divided to explore the changes of heterogeneity and treatment effects. The results were demonstrated in a forest plot (Figure 9). Studies testing on Low-back and postsurgical pain had a low heterogeneity with *I^2^* = 0% and 23% respectively. Studies testing on Arthritis and Back also had a moderate heterogeneity with *I^2^* = 55% and 63% respectively. Common effects models and Random effects models were built for subgroups based on if *I^2^* < 50% or not.

It showed that groups with participants experiencing chronic lower back and back pain had insignificant treatment results with 95% CI covering 0. Other subgroups had significant treatment effects with a negative mean difference substantially lower 0 and 95% CI without covering 0. It means that patients in these Age groups taking NSAID drugs obtained significant pain reduction compared to those who took Placebo. As presented in Figure 9, NSAID drugs had over 1 point pain reduction on patients with neuropathic (-1.90), arthritis (-1.13) and other unknown types of chronic pain (-1.10). While for patients with postsurgical pain, the pooled pain reduction effect decreased to 0.42 under a common effects model. Drugs used for different chronic pain had different treatment effects and the heterogeneity was not stable in certain subgroups. It indicated that Pain type should be considered when dealing with heterogeneity and estimation bias.

**Network Meta-analysis (NMA)**

A NMA [Figure 5] was completed for 34 studies. The nodes correspond to each intervention included within the network where the interventions with direct comparisons are linked with a line. The thickness of lines corresponds to the number of trials evaluating the comparison. A connected network was built based on the placebo which was mostly Tolterodine based on the original studies. The evaluations between interventions were supported by direct comparison and indirect comparison.

*Subgroup analysis for Pain Score Difference based on different geographical locations*

Using the geographical locations of the studies, a subgroup analysis was conducted and demonstrated in a forest plot (Figure 11). A statistically significant difference (p-value < 0.01) was identified between different subgroups with a respectively higher or lower pooled mean difference compared to the overall value in PMA. The pooled treatment results were better in Asia (MD = -1.56, 95% CI = [-2.34, -0.78]) and Europe ( MD = -1.20, 95% CI = [-1.85, -0.55]) with the pain reduction exceeding 1 point scale. However, for studies conducted in Oceania had a decreased treatment effect with a mean difference of -0.64 (95% CI = [-1.26, -0.03]). And studies conducted in North America had insignificant treatment results as the 95% CI [-0.69, 0.42] of mean difference (-0.13) contained 0. It also showed that heterogeneity changed in groups Oceania (*I^2^* = 0%, p-value = 0.96), Europe (*I^2^* = 70%, p-value = 0.02), and North America (*I^2^* = 75%, p-value < 0.01), indicating that geographical locations might be one influential source of heterogeneity in our analysis.

*Sub-group analysis based on pain type*

As for pain types, NSAID drugs had over 1-point pain reduction on patients with neuropathic (-1.90), arthritis (-1.13) and other types of chronic pain (-1.03). While for patients with postsurgical pain, the pooled pain reduction effect decreased to 0.42 under a common effects model. Studies  testing on Low-back and postsurgical pain had a low heterogeneity with *I^2^* = 0% and 23% respectively. Studies exploring arthritis and chronic back pain also had a moderate heterogeneity with *I^2^* = 55% and 63% respectively. Studies exploring neuropathic pain and other unknown types of chronic pain had a high heterogeneity with *I^2^* = > 90% .

A high heterogeneity of 94% and 87% of (p-value < 0.01) was identified in studies with study periods lasting for months and weeks, respectively (Figure 10). Long term follow-up time was common among RCTs although the periods different and attributed to heterogeneity by involving a variety of covariates, assessment tools and other factors.

After grouping based on testing intervals, studies for months had a better treatment effect with a mean difference of -1.05 (95% CI = [-1.77, -0.33]) compared to a placebo. Studies using weeks (-0.83) and hours (-0.47) had significant results but decreased the absolute values of pain reduction. An insignificant pain reduction in group of Minutes with 95% CI covering 0 (MD = -0.47, 95% CI = [-1.09, 0.11]). This is more relevant to studies reporting on post-surgical pain as pain management is often more aggressively provided short term and then curbed or stopped after 7 to 14 days.
